# A compendium of expression patterns of cholesterol biosynthetic enzymes in the mouse embryo[S]

**DOI:** 10.1194/jlr.M059634

**Published:** 2015-08

**Authors:** Melda Şişecioğlu, Harun Budak, Lars Geffers, Murat Çankaya, Mehmet Çiftci, Christina Thaller, Gregor Eichele, Ömer İrfan Küfrevioğlu, Hasan Özdemir

**Affiliations:** * Departments of Molecular Biology and Genetics Faculty of Science, Ataturk University, 25240 Erzurum, Turkey; ** Chemistry, Faculty of Science, Ataturk University, 25240 Erzurum, Turkey; † Genes and Behavior Department, Max Planck Institute of Biophysical Chemistry, 37077 Goettingen, Germany; § Department of Biology, Faculty of Arts and Sciences, Erzincan University, 24100 Erzincan, Turkey; †† Department of Chemistry, Faculty of Arts and Sciences, Bingol University, 12000 Bingol, Turkey

**Keywords:** in situ hybridization, brain lipids, cholesterol/biosynthesis, eye, gene expression, nutrition, signal transduction, Smith-Lemli-Opitz syndrome

## Abstract

Cholesterol and its biosynthetic pathway intermediates and derivatives are required for many developmental processes including membrane biogenesis, transmembrane receptor signaling, steroid biogenesis, nuclear receptor activation, and posttranslational modification of hedgehog (Hh) proteins. To perform such multifaceted tasks depends on stringent regulation of expression of cholesterol biosynthetic enzymes (CBEs). We established for a whole organism, for the first time, the 3D expression pattern of all genes required for cholesterol biosynthesis (CBS), starting from acetyl-CoA and ending with cholesterol. This data was produced by high-throughput in situ hybridization on serial sections through the mouse fetus. The textually annotated image data were seamlessly integrated into the METscout and GenePaint public databases. This novel information helps in the understanding of why CBEs are expressed at particular locations within the fetus. For example, strong CBE expression is detected at sites of cell proliferation and also where cell growth increases membrane surface, such as in neurons sprouting axons and forming synapses. The CBE data also sheds light on the spatial relationship of cells and tissue that express sonic Hh (Shh) and produce cholesterol, respectively. We discovered that not all cells expressing Shh are capable of CBS. This finding suggests novel ways by which cholesterylation of Shh is regulated.

Embryogenesis is characterized by significant mass increase which is caused by cell proliferation and cell growth, both of which depend on continuous production of cellular membranes. Because such membranes contain up to 20 weight percent cholesterol, availability of this compound is essential for embryogenesis. Cholesterol maintains membrane integrity and also contributes to and regulates the properties of lipid rafts and lipid microdomains. Rafts and microdomains are the sites where many transmembrane signaling processes occur that play pivotal roles in development (1, 2). Cholesterol also has very direct effects. It is covalently bound to the N-terminal fragment of hedgehog (Hh) proteins, and has essential roles in pattern formation and morphogenesis in invertebrates and vertebrates. The C-terminally attached cholesterol is required for the formation of multimeric Hh complexes and is thought to regulate the distribution of extracellular Hh ligand (3). Furthermore, cholesterol is a precursor of steroid hormones, bile acids, and oxysterols, each of which binds to specific nuclear receptors, which are potent transcriptional activators that control a wide spectrum of developmental and physiological processes (4–6).

The origin of cholesterol is twofold. First, in the adult, cholesterol is taken up from the diet; whereas in the embryo, cholesterol originates from the maternal circulation (1, 2, 7). Second, cholesterol is de novo synthesized by adult and embryonic tissues along a reaction pathway encompassing >30 different steps catalyzed by >20 cholesterol biosynthetic enzymes (CBEs) located in cytosol, endoplasmic reticulum, mitochondria, and peroxisomes (8). The multistep cholesterol synthesis partitions into the presqualene and postsqualene segments (supplementary Fig. 1). Squalene synthesis initiates with acetyl-CoA and leads to lanosterol, at which the pathway bifurcates into the Bloch and Kandutsch-Russell pathways, respectively. Both of these pathways eventually lead to cholesterol.

The most compelling evidence for a pivotal role of cholesterol biosynthesis (CBS) for proper mammalian development comes from human and mouse genetic mutations in CBE genes (2, 9). The first human inborn error subsequently associated with a defective cholesterol metabolism is the Smith-Lemli-Opitz syndrome (SLOS) (10). SLOS is an autosomal recessive disorder caused by mutations in the *DHCR7* gene that encodes 7-dehydrocholesterol reductase converting 7-dehydrocholesterol to cholesterol (supplementary Fig. 1) (11, 12). The phenotypic spectrum of SLOS is very broad, ranging from death in utero to major malformations (skeletal, congenital heart, lung, and kidney defects) to mild cases with minor physical defects and impaired learning and behavior. The essentiality of cholesterol for normal mammalian development is further emphasized by the finding that mutations in other human CBE genes often evoke strong phenotypes, albeit sometimes different ones, for each gene (2, 9). Additionally, many mutations in murine CBE homologs evoke strong phenotypes that are, at least in part, reminiscent of those seen in *Homo sapiens*.

Although cholesterol deficiency is a hallmark of all these disorders, it has been proposed that accumulating cholesterol synthesis intermediates and their metabolites also contribute to the disease phenotypes. Knowing when in development and in which organ a particular *CBE* is expressed thus helps in understanding the physiology and pathophysiology of CBE disorders. More broadly, such data sheds light on the spatiotemporal dynamics of cholesterol metabolism in the context of other metabolic and signaling pathways, puts *CBE* expression into the context of its upstream regulators such as sterol regulatory element-binding proteins (SREBPs), and can lead to the identification of sites of accumulation of potentially toxic intermediates and metabolites of CBS. As a contribution toward advancing knowledge in these areas, we have determined the expression patterns of the 25 genes encoding CBEs using high-throughput in situ hybridization (ISH). CBE gene expression was analyzed to conform with and match to the ISH data of the transcriptome-wide GenePaint and EURExpress digital atlases (13, 14) that house thousands of gene expression patterns, including those for the Hh signaling pathway and for the steroid-, oxysterol-, and bile acid-controlled gene transcription networks. Additionally, CBE gene expression data were integrated into the METscout database, a powerful database in which cholesterol metabolism is integrated into the global metabolic network of the mouse (15).

## MATERIALS AND METHODS

### DNA template production and RNA probe synthesis

DNA template production and RNA probe synthesis were performed using previously described procedures (16, 17). Template sequences were deposited on the GenePaint database and can be retrieved on the set viewer page for each of the genes examined.

### Tissue collection, sectioning, and ISH

The general method for the dissection of E13.5 or E14.5 embryos, tissue collection, and tissue sectioning, fixation, and ISH was performed as previously reported (16, 17). The E14.5 embryo expression pattern for each gene is documented in 22–24 sagittal sections.

### Imaging and data management

The detailed procedures were previously described (17). Images were uploaded to the GenePaint and METscout databases (13, 15, 16).

### Databases

**Fig. 1.**
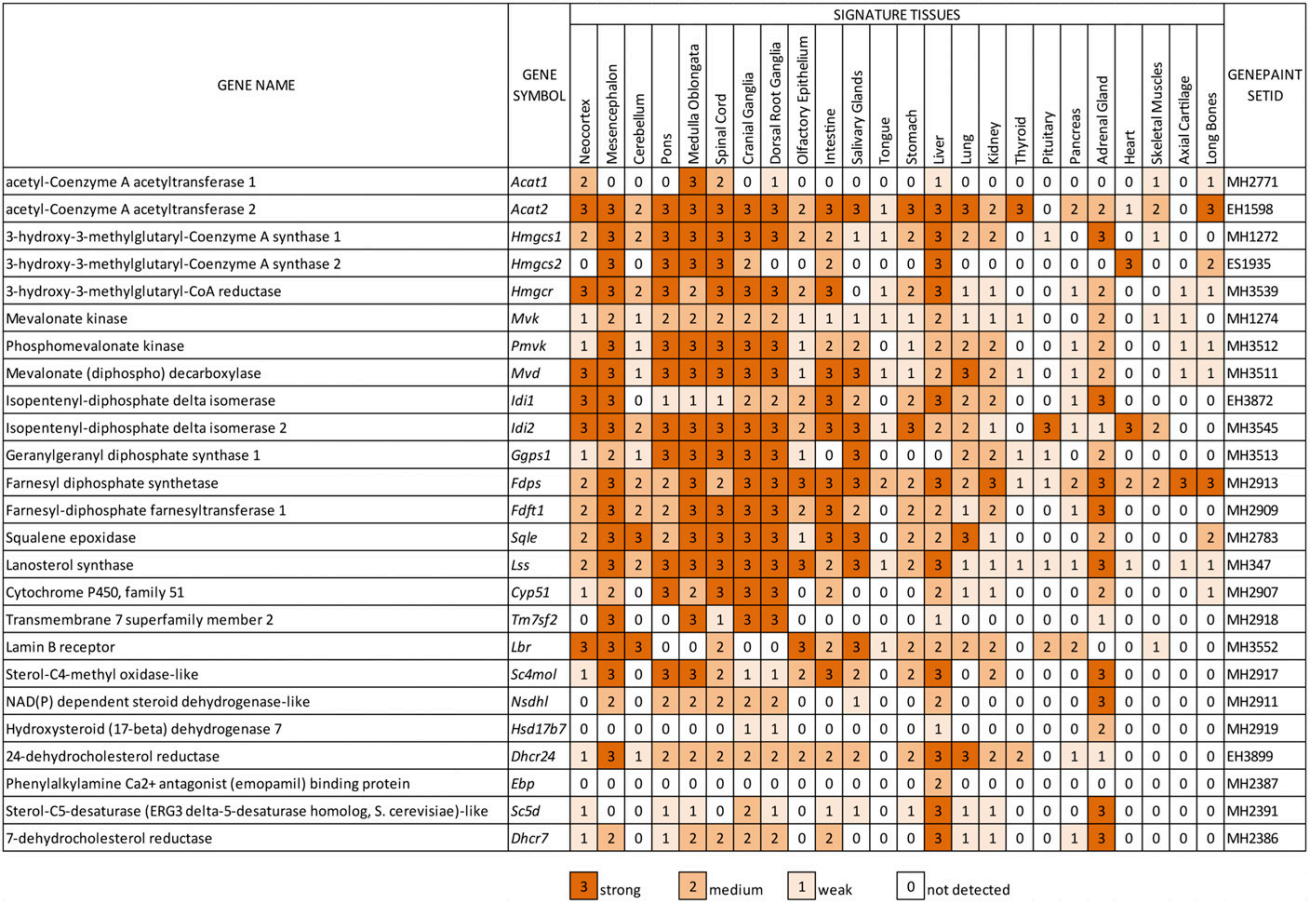
Summary diagram of *CBE* expression patterns in 24 signature tissues. For each tissue the expression level (strong, medium, weak, and not detected) of each transcript of the 25 CBEs is shown in shades of caramel. The GenePaint set ID in the rightmost column identifies the ISH reference data set which can be fully viewed at GenePaint or METscout.

All primary ISH data on which this publication is based are accessible on the METscout database by typing the gene symbol (provided in the second column of Fig. 1) into the gene entry field found on the front page. For a description of METscout see (15). Additionally, data were uploaded onto GenePaint and are accessed therein using the GenePaint set ID found in the rightmost columns of Fig. 1 and supplementary Table 1.

## RESULTS

### CBE mRNAs reveal a stereotypical energy metabolism pattern

The spatial expression profiles of 25 genes encoding CBEs (see supplementary Table 1 for a listing of components and supplementary Fig. 1 for the pathway) were determined at stage E14.5 of mouse embryonic development by ISH. At this stage of development, organs begin to differentiate and some of them have started to carry out their particular metabolic and/or endocrine functions. Additionally, embryos undergo exponential growth and hence depend extensively on cellular membrane biosynthesis. E14.5 is also a time in development where many of the developmental signals such as Hh are still highly active.

ISH results are summarized in the color chart in **Fig. 1** in which the level of expression of mRNAs encoding the CBEs is shown for a series of signature tissues. The strongest expression is observed in the central nervous system, in the liver, and in the adrenal glands. It is evident that the levels of expression of the pathway components differ quantitatively in any given tissue. Moreover, a particular *CBE* transcript shows considerable variation across the tissue spectrum, reminiscent of the situation in adult tissues in which some cells produce cholesterol in excess while others take up cholesterol. Although not every tissue expresses the full complement of CBE, this would not imply that cholesterol synthesis is absent in those tissues because precursors are found in and exchanged via the bloodstream. At E14.5, blood circulation is functional such that tissues have access to blood-borne metabolites. Also note that ISH data represents mRNAs of CBEs, which are predictive of expression of the cognate proteins. However, it is widely appreciated that levels of mRNA and of protein activity are not always proportional. The data shown in the color chart can readily be confirmed by a visual inspection of the comprehensive ISH primary data on METscout or GenePaint.

**Fig. 2.**
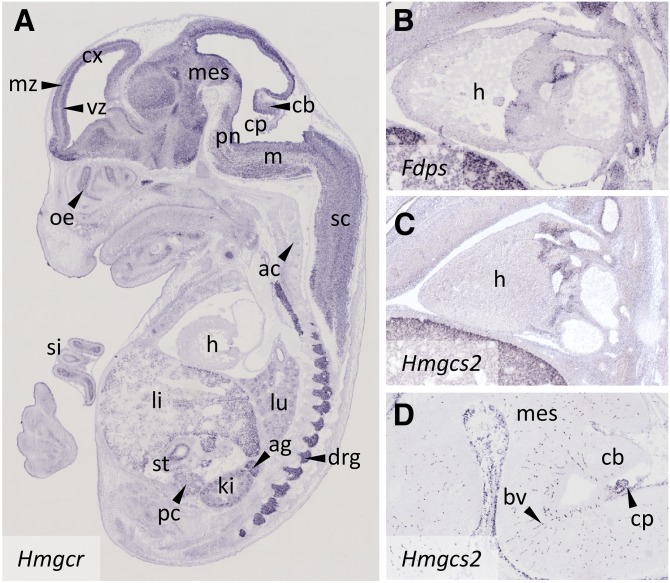
*CBE*s exhibit a SEMP in the E14.5 mouse embryo. A: *Hmgcr* expression is emblematic for a SEMP, except for the absence of expression in the heart. B, C: *Fdps* and *Hmgcs2* are the only *CBE*s detected in the developing heart. D: In contrast to other *CBE*s, transcripts of *Hmgcs2* localize predominantly to blood vessels passing through the brain parenchyma, meninges, and choroid plexus. ag, adrenal gland; ac, axial cartilage; bv, blood vessels; cb, cerebellum; cp, choroid plexus; cx, neocortex; drg, dorsal root ganglion; h, heart; inbl, inner neuroblastic layer; iz, intermediate zone; ki, kidney; li, liver; lu, lung; m, medulla oblongata; mes, mesencephalon; mz, marginal zone; ob, olfactory bulb; oe, olfactory epithelium; onbl, outer neuroblastic layer; pc, pancreas; pn, pons; sc, spinal cord; si, small intestine; st, stomach; vz, ventricular zone; vf, vibrissae follicles.

The distribution of expression strength of CBE mRNAs across signature tissues resembles that of the “stereotypical energy metabolism pattern” (SEMP) that characterizes glycolytic and TCA cycle components (18). For example, expression of *Hmgcr* encoding the rate-limiting 3-hydroxy-3-methylglutaryl-CoA reductase varies considerably across the signature tissues (Fig. 1, **Fig. 2A**). There is one marked difference with SEMP: *CBE*s are mostly not detectable in the heart (Figs. 1, 2A), except for *Fdps* and *Hmgcs2*, whose transcripts are found in the valves of the developing heart and the developing blood vessels (*Hmgcs2* only) (Fig. 2B–D). Elevated, but variable, expression of *CBE*s is seen in the mesencephalon, the pons, the medulla, the dorsal root ganglia, the cranial ganglia, the liver, the adrenal, and the kidney (Figs. 1, 2A).

**Fig. 3.**
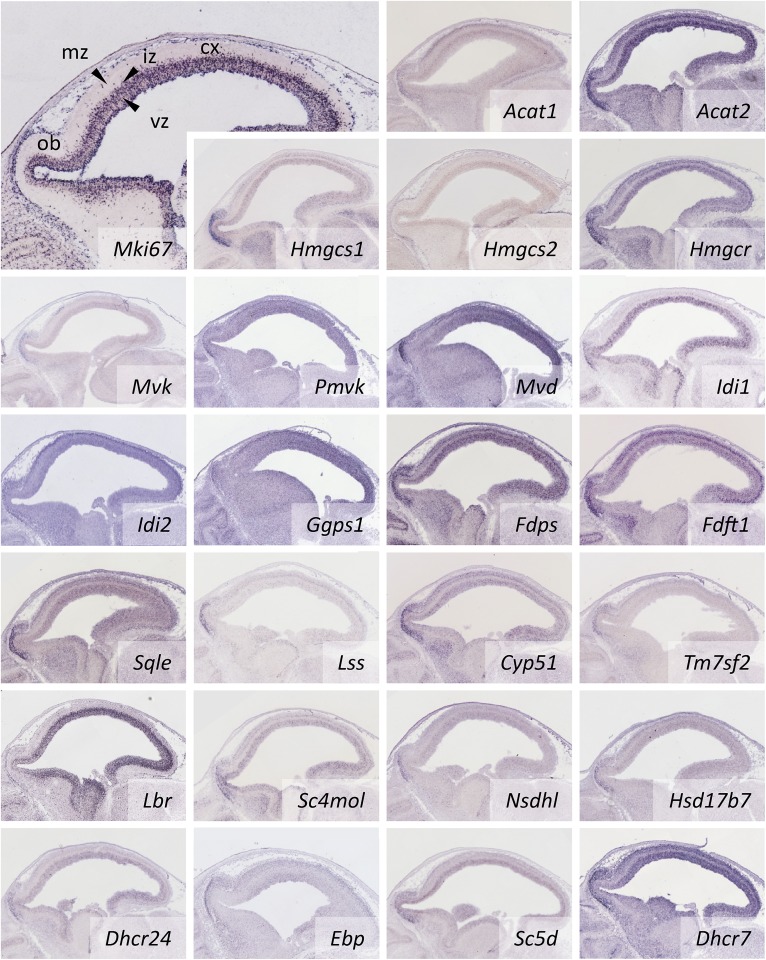
Expression of *CBE*s in the developing forebrain. In all cases *CBE* transcripts are found in the ventricular zone that houses proliferating neuronal progenitor cells (*Mki67* ISH image, top left) but frequently also in regions with postmitotic cells, such as the marginal zone and a crescent-shaped domain in the olfactory bulb anlage. For abbreviations see the Fig. 2 legend.

Most CBEs are strongly expressed in the developing cerebral cortex (**Fig. 3**). The innermost cortical layer (at this developmental stage referred to as the ventricular zone) contains the cell bodies of mitotic cells that express the mitotic marker, *Mki67* (Fig. 3, top left); whereas the intermediate and marginal zones are mostly populated by postmitotic neuronal progenitors. The intermediate zone expresses very low levels of *CBE*s while the two other zones express *CBE*s. Additionally, most *CBE*s are strongly expressed in a semi crescent-shaped domain extending along the outer boundary of the olfactory bulb (Fig. 3). Apparently proliferating neuronal progenitors in the ventricular zone require cholesterol for making more cell membrane in the process of spinning off daughter cells. The need for cholesterol in postmitotic neurons of the marginal zones in the cortex and the olfactory bulb could reflect cell growth and, in particular, the formation of neuronal networks that begin to emerge in these regions. Establishment of these networks depends on cholesterol too, as there is a substantial increase of the cell surface and synaptic connections are formed (19).

**Fig. 4.**
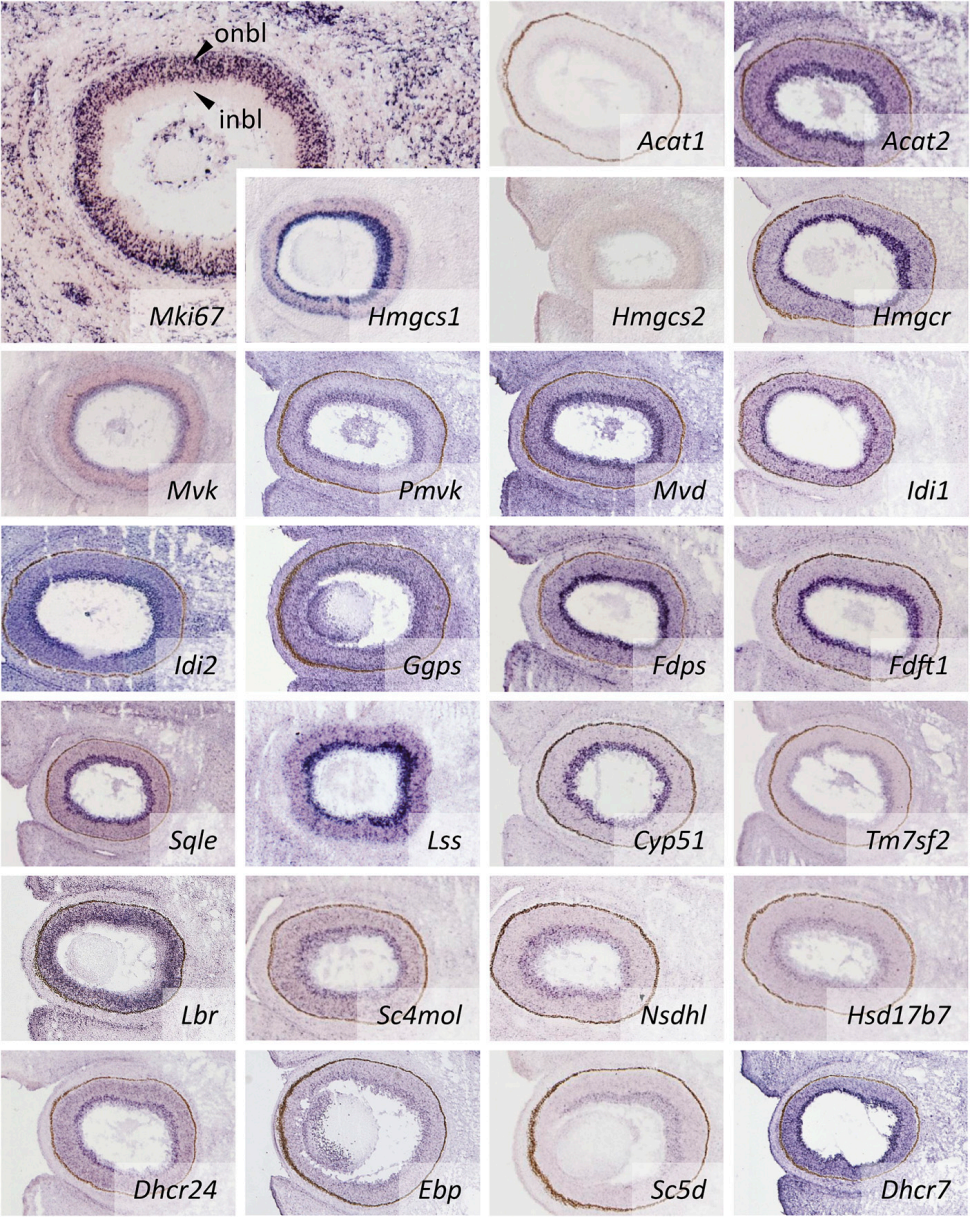
Expression of *CBE*s in the developing eye. Transcripts of all *CBE* genes are detected in the inner neuroblastic layer which contains postmitotic neuronal progenitors that do not express the proliferation marker *Mki67* (see ISH picture top left). For abbreviations see the Fig. 2 legend.

The embryonic retina contains both proliferating neuronal precursors (outer neuroblastic layer, expressing the proliferation marker Mki67; **Fig. 4**, top left) and postmitotic growing neurons (inner neuroblastic layer). Interestingly, most mRNAs encoding CBEs are detected in the inner neuroblastic layer, i.e., in postmitotic cells that undergo differentiation, which is, as noted for the marginal zone of the cortex, accompanied by a marked increase in the membrane surface due to the formation of axons of the optic tract plus the formation of synaptic connections.

Apart from its contribution to the formation of cellular membranes, cholesterol might be required by mitotic cells of the developing cortex in lipid raft microdomains that contain transmembrane signaling proteins, such as growth factor receptors. For example, the FGFR1 and -2 receptors are expressed in the ventricular zone (GenPaint set ID EH130 and FG35) and FGFR2 is present in rafts (20).

### CBEs in the adrenal glands

**Fig. 5.**
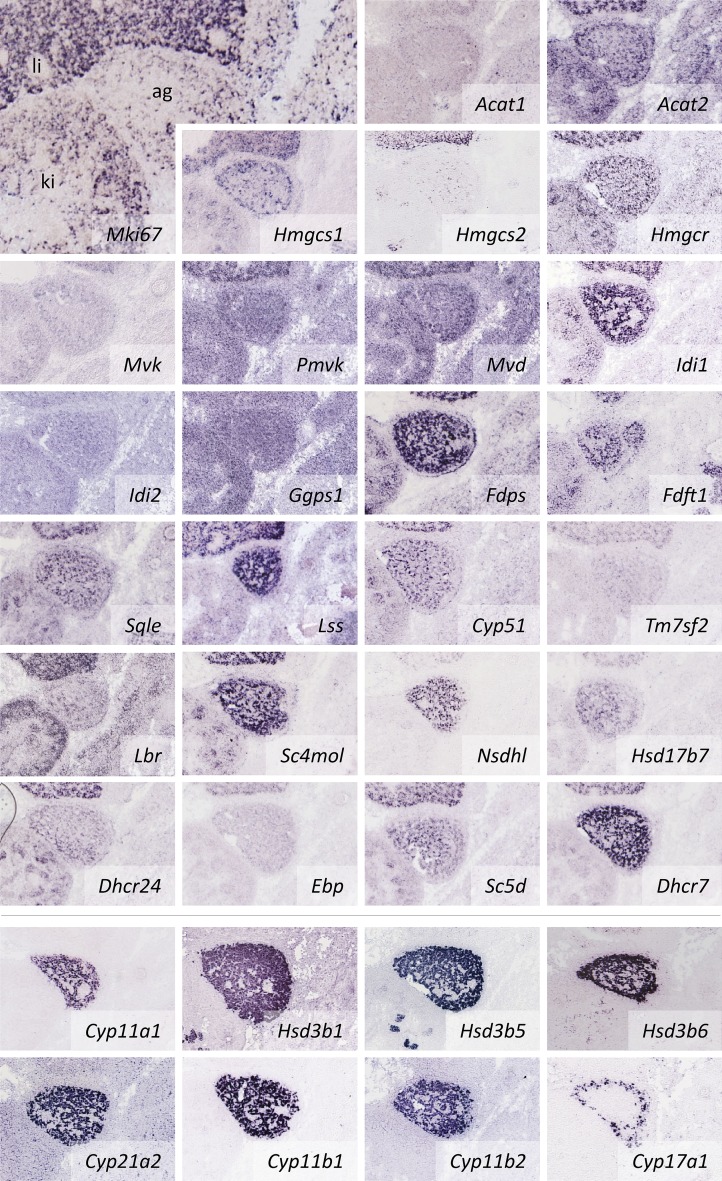
Expression of *CBE*s in the developing adrenal gland. All components of the CBS pathway are represented by at least one expressed isoenzyme in the developing adrenal gland. Expression of *CBE*s does not necessarily overlap with proliferating cells (*Mki67* ISH image top left) but is in strong agreement with the expression patterns observed for steroid hormone synthesis (bottom rows separated by a line). For abbreviations see the Fig. 2 legend.

The components of the CBS pathway are expressed in the adrenal gland, even at embryonic stage E14.5 (Fig. 1, **Fig. 5**). In this tissue, cholesterol serves as a precursor for adrenal steroid biosynthesis (supplementary Fig. 2). The six catalytic activities required for the synthesis of glucocorticoids, mineralocorticoids, and the sex hormone precursor, androstenedione, are all expressed in the developing adrenal. Of the five *Hsd3b* isozymes (*Hsd3b1*, *-2*, *-4*, *-5*, and *-6*), *Hsd3b1*, *-5*, and *-6* are expressed. Expression patterns of *Cyp*s and *Hsb3*s are nearly uniform, except for *Cyp17a1* that is predominantly found along the margin of the adrenal and is required for androstenedione and cortisol synthesis. Therefore, a medulla/cortex regionalization in steroid synthesis can be seen already during development.

### CBS along midline structures

**Fig. 6.**
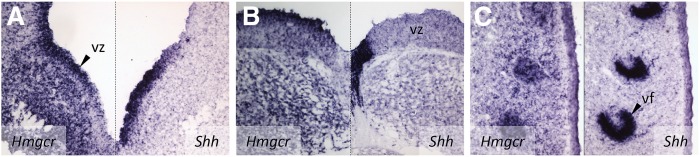
Expression domains of *Hmgcr* and *Shh* in the E13.5 mouse embryo. The ventricular zone of the hypothalamic area (A), the mesencephalic area (B), and the vibrissae follicles (C) of the snout illustrate in contralateral (A, B) and ipsilateral (C) adjacent coronal sections that the expression domains of *Hmgcr* and *Shh* show substantial, but not complete, overlap. For abbreviations see the Fig. 2 legend.

Cholesterol is conjugated to Hh proteins [including sonic Hh (Shh)], which raises the question about the relative relationship of the expression domains of *Shh* and the *CEB* mRNAs. In contrast to *CEB*s, the *Shh* gene is expressed in a highly restricted manner, often in signaling regions that control pattern formation and morphogenesis in the surrounding tissues. The expression pattern of *Shh* in the developing brain is highly dynamic (21). A sagittal section through the midline of an E14.5 embryo reveals a prominent expression domain of *Shh* in the shape of a narrow band found on the floor of the midbrain and extending into the hypothalamic region of the forebrain (GenPaint set ID MH459, midline sagittal section). As judged from midline sagittal sections through E14.5 embryos subjected to ISH with *CBE* probes, the majority of *CBE*s replicate this pattern. For the majority of *CBE*s, elevated expression is detectable along the mesencephalic and telencephalic midline, with decreasing strength in the more lateral neuronal tissue (see e.g., the SLOS-causative gene *Dhcr7*, GenePaint set ID MH2386). To fully capture the overlap between the expression domains of *CBE*s and *Shh*, cross-sections through the floor of the hypothalamic and mesencephalic regions of an E13.5 embryo were prepared. Extending previous work (21), hybridization with a *Shh* probe revealed a V-shaped expression domain of *Shh* that coincides with the ventricular zone (**Fig. 6A**, right). *Hmgcr*, the rate-limiting regulatory enzyme of the pathway, is broadly expressed in the wall of the hypothalamus, but the strength of expression is markedly upregulated in the ventricular zone (Fig. 6A, left). Taken together, there is, within the ventricular zone, a substantial but not complete overlap of expression of *Shh* and *Hmgcr*. The transverse sections through the floor region of the mesencephalon (Fig. 6B) show restricted expression of *Shh* in a ventralmost region and widespread expression of *Hmgcr* throughout the nervous tissue, peaking in cells adjacent to the expression domain of *Shh*. Therefore, the data shown in Fig. 6B emphasize that there is but a limited overlap between the two expression domains. Figure 6C illustrates the expression domains of *Shh* and *Hmgcr* in the primordium of the fascial vibrissae. The sickle-shaped expression domain of *Shh* is substantially overlapping with that of *Hmgcr*. It is obvious that for forming a covalent adduct, SHH and cholesterol have to colocalize and, at least as judged on the localization of *Shh* and *Hmgcr* transcripts, this is not always the case. In the case shown in Fig. 6A coexpression is limited to a specific region, which could potentially serve as a very restricted, and hence precisely defined, source of cholesteryl-Shh. In the situations exemplified in Fig. 6B, there is virtually no overlap between the expression domains, but exogenous cholesterol present in cerebrospinal fluid filling the ventricles could act as a cholesterol source. This exogenous cholesterol could also be of maternal origin.

## DISCUSSION

Although open access ISH data have previously been used to characterize cholesterol homeostasis in the mouse hippocampus (22), the present study is the first one to provide a comprehensive view of the spatial organization of CBS for a whole organism. From the wealth of data present in the *CBE* expression atlas, three aspects were selected that shed light on and are relevant for cholesterol’s physiological roles: *i*) in the biogenesis and stability of membranes, *ii*) as a precursor for adrenal steroids; and *iii*) as an essential covalent modifier of Hh proteins.

The expression patterns of CBEs strongly resemble those of enzymes mediating cellular energy metabolism that constitute the Krebs cycle and glycolysis. These enzymes, like the CBEs, provide building blocks for cellular structures. In the case of the rapidly growing embryo, such building blocks are constantly and ubiquitously required. Nonetheless, neither TCA nor glycolytic enzymes and CBEs are uniformly expressed, but there are substantial differences in expression levels between and within tissues. Emblematically, *CBE* expression levels in developing neocortex and retina were examined. Both tissues are characterized by the presence of proliferating and postmitotic differentiating neurons. We found that the highest levels of *CBE* expression in the retina are found in the inner neuroblastic layer, devoid of any substantial cell proliferation, but characterized by neuronal differentiation tied to growing neurites that eventually have to reach distant targets in the brain. In addition, the developing retina is characterized by the formation of synaptic connections. Both axon genesis and synapse formation require the formation of new membrane bilayers. In the neocortex, the highest *CBE* levels are seen in the ventricular zone in which cells divide at high rates and, therefore, membrane synthesis is critical in the ventricular zone. Additionally, neuronal progenitors of the ventricular zone receive growth factor signals from the cerebrospinal fluid-filled ventricle. These factors bind to their cognate transmembrane receptors, some of which are organized in cholesterol-containing lipid rafts.

It is interesting to compare the *CBE* expression data with measurements of total sterols in the mouse embryo (23). Based on a comparative analyses of *Dhcr7* knockout (a SLOS model) and wild-type mice, it was concluded that in the brain, cholesterol is chiefly of maternal origin until about E10–E11. In liver and lung, the dam provides cholesterol until about E12–E14. These data are in strong agreement with our ISH data that definitely show the presence of expression of *CBE*s by E14.5.

The present study also clarifies the spatial relationship of the localities of CBS and of *Shh* expression. As a proxy for the former, the expression of *Hmgcr* was used, because this gene encodes a key regulatory enzyme of CBS. The most important finding in this part of the study is that *Shh* and *Hmgcr* are overlapping, but not always and not completely; we show cases in which expression domains of the two are adjacent. In this situation, one would hypothesize that cholesterol would have to be taken up by *Shh*-expressing cells either from neighboring tissues that synthesize this compound or from cerebrospinal fluid that fills the space of the brain ventricles.

Because the expression patterns of *CBE*s seamlessly fit into the data deposited on the GenePaint and EURExpress databases, the *CBE* data can readily be integrated into that body of transcriptome-scale data. We illustrate this point by combining expression data for *CBE*s with those of the enzymes that convert cholesterol to adrenal steroids. This way, the complete pathway starting from acetyl-CoA could be reconstructed showing that even embryonic adrenals are equipped with all the components required for the synthesis of all adrenal steroids.

Many of the CBE-encoding genes are characterized by sterol regulatory elements, which are binding sites for SREBP1 and SREBP2 (8). The transcripts (*Srebp1* and *-2*) for both proteins show expression patterns reminiscent of those of the *CBE* genes. Strong expression of *Srebp1* is seen in the ventricular zone of the forebrain, in the liver, and in the *Shh*-positive midline of the midbrain (GenPaint set ID MH946). *Srebp2* transcripts are also found in the ventricular zone and in the midbrain, but to a lesser extent in the liver. Furthermore, *Srebp2*, but not *Srebp1*, is strongly expressed in the peripheral nervous system. It appears that CBE regulators *Srebp1* and *Srebp2* are characterized by both coexpression in some tissues and complementary expression in others.

Human congenital disorders tied to cholesterol metabolism and the corresponding mouse models have strong embryonic phenotypes [for a review see (2)]. At least to some extent, knowing all sites of expression of CBEs can rationalize developmental abnormalities and, hence, guide further research. Thus the digital compendium of *CBE* expression patterns in conjunction with expression data for genes up- and downstream of CBS provides a most useful resource for the interpretation of biological, genetic, and disease data.

## Supplementary Material

Supplemental Data
