# Supplementary material for: A compendium of expression patterns of cholesterol biosynthetic enzymes in the mouse embryo
Source: J Lipid Res. 2015 Aug;56(8):1551–9. doi: 10.1194/jlr.M059634 (PMC4513996; doi:10.1194/jlr.M059634)
Supplement: Supplemental Data [file supp_56_8_1551__index.html]

A Compendium of Expression Patterns of Cholesterol Biosynthetic Enzymes in the Mouse Embryo — A compendium of expression patterns of cholesterol biosynthetic enzymes in the mouse embryo — Supplemental Data 

# A compendium of expression patterns of cholesterol biosynthetic enzymes in the mouse embryo

## Supplemental Data

- Supplemental Fig. S I -IV - Supplemental Figure S I: Synthesis of cholesterol. Supplemental Figure S II: Synthesis of adrenal steroid hormones. Supplemental Figure S III: Gene expression patterns of Scarb1. Supplemental Figure S IV: Gene expression patterns of Ldlr.
- Supplemental Table S I - Supplemental Table S I: Enzymes in Cholesterol Biosynthesis.
