# Supplementary material for: A compendium of expression patterns of cholesterol biosynthetic enzymes in the mouse embryo
Source: J Lipid Res. 2015 Aug;56(8):1551–9. doi: 10.1194/jlr.M059634 (PMC4513996; doi:10.1194/jlr.M059634)

Supplemental Figure legends

<u>Supplemental Figure S I:</u> Synthesis of cholesterol. The subcellular organization of the CBS pathway is shown with all CBE genes (bold italics) listed in Fig. 1. For each encoded enzyme the direction of reaction is indicated (brown arrow/-head). Subcellular compartments (blue, boxed text) are demarcated with blue boundaries. Pathways connected to CBS are colored in slate blue. Metabolites are color-coded to distinguish the presqualene (light orange) from postsqualene (dark orange) segment which bifurcates at the level of lanosterol into the Bloch (turquois) and Kandutsch-Russell (purple) pathway leading to cholesterol (old rose). Transport between compartments is indicated by black arrows. Green boxes highlight the contribution of CBS to important products based on farnesyl-PP, 7-dehydrocholesterol, and cholesterol. **Abbreviation:** HMG, 3-hydroxy-3-methylglutaryl

<u>Supplemental Figure S II:</u> Synthesis of adrenal steroid hormones. The spatial organization of steroid hormone synthesis as it occurs in the mature adrenal cortex is shown with all genes depicted in Fig. 5 below the dashed line. Boxed text (blue) indicates the zones of the mature adrenal cortex. The scheme is uses the same general layout as Supplemental Fig. 1 and continues from Cholesterol.

<u>Supplemental Figure S III+IV:</u> Gene expression patterns of *Scarb1*and *Ldlr*. *Scarb1* and *Ldlr* encode receptors involved in cholesterol uptake.

Supplemental Figure S I: Synthesis of cholesterol.

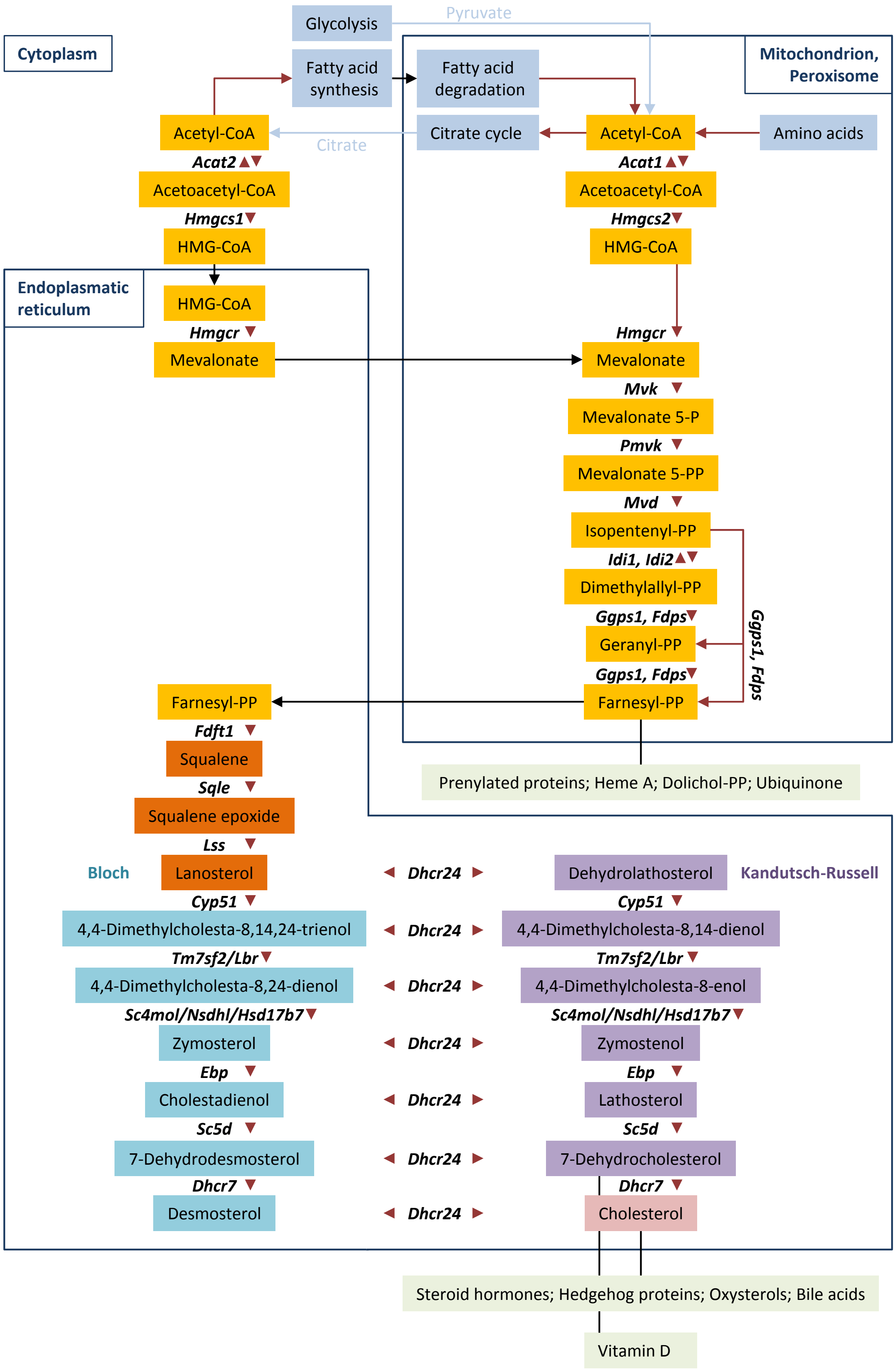

Supplemental Figure S II: Synthesis of adrenal steroid hormones.

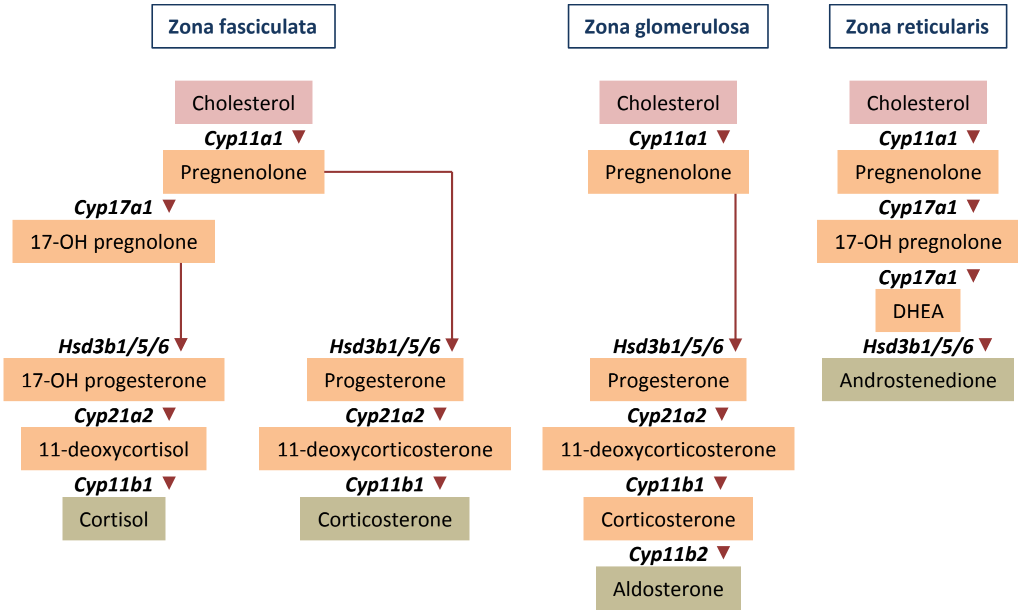

Supplemental Figure S III: Gene expression pattern of *Scarb1*.

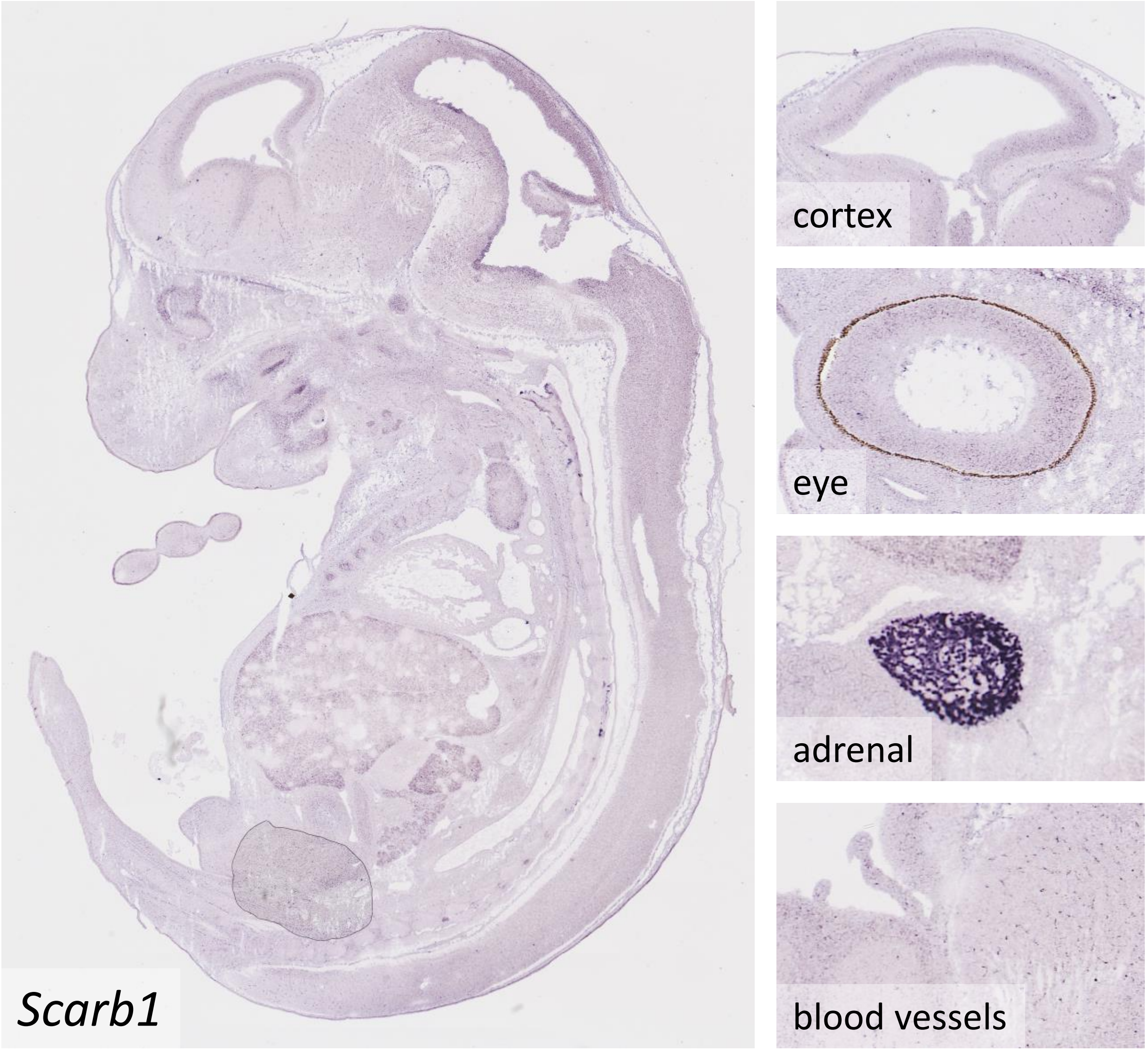

Supplemental Figure S IV: Gene expression pattern of *Ldlr*.

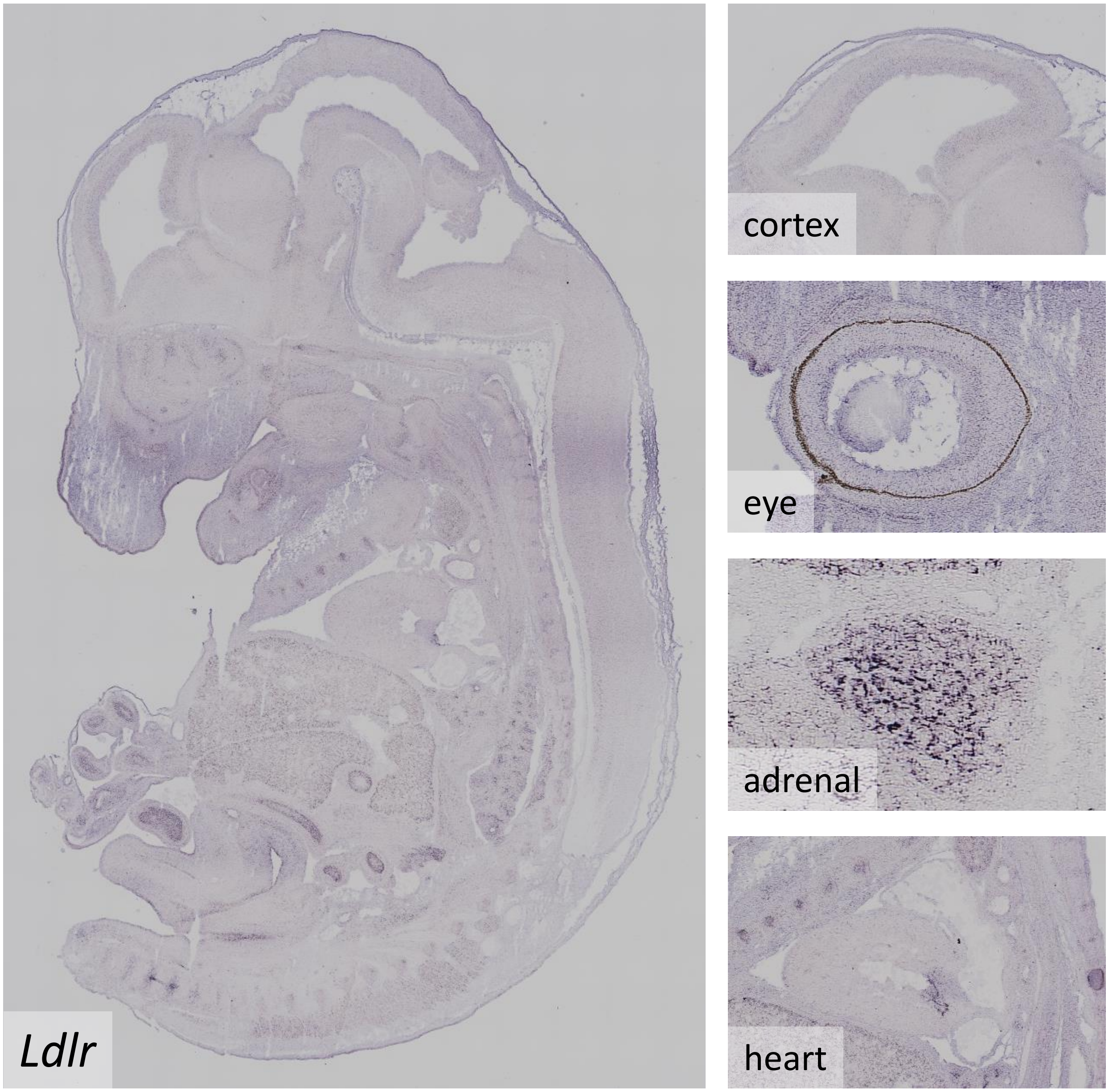

Supplement: Supplemental Data [file supp_M059634_jlr.M059634-1.pdf]
